# Supplementary material for: Epidemiology of respiratory syncytial virus in young, hospitalized children in Jordan: a prospective viral surveillance study
Source: Microbiol Spectr. 2025 Oct 15;13(11):e01727-25. doi: 10.1128/spectrum.01727-25 (PMC12584765; doi:10.1128/spectrum.01727-25)
Supplement: Supplemental material — Table S1; Fig. S1; Jordan Viral Surveillance Studies Group member list. [file spectrum.01727-25-s0001.docx]

# Supplemental Material

**Table S1.** Common respiratory virus detections among children hospitalized at Al-Bashir Hospital (Amman, Jordan) with fever or respiratory symptoms between January 8, 2023, and April 30, 2024.

| **Virus** | **Detections, *n* (%)** | |
| --- | --- | --- |
|  | **Children <5 years old, *N*=2,610** | **Children <2 years old, *n*=2,265** |
| HRV | 733 (28.0) | 639 (28.2) |
| RSV | 713 (27.3) | 680 (30.0) |
| AdV | 325 (12.4) | 255 (11.2) |
| HMPV | 255 (9.8) | 218 (9.6) |
| PIV | 216 (8.3) | 193 (8.5) |
| ccCoV | 207 (7.9) | 194 (8.6) |
| SARS-CoV-2 | 192 (7.3) | 185 (8.2) |
| Flu | 177 (6.8) | 127 (5.6) |

**Abbreviations:** HRV, human rhinovirus; RSV, respiratory syncytial virus; AdV, adenovirus; HMPV, human metapneumovirus; PIV, parainfluenza virus; ccCoV, common cold coronavirus; SARS-CoV-2, severe acute respiratory syndrome coronavirus 2; Flu, influenza virus.

**Figure S1.** Respiratory syncytial virus (RSV) detection status among subgroups of children (defined by age group at admission) hospitalized at Al-Bashir Hospital (Amman, Jordan) with fever or respiratory symptoms between January 8, 2023, and April 30, 2024. (a) Relative frequencies of RSV detection status. (b) Absolute frequences of RSV detection.


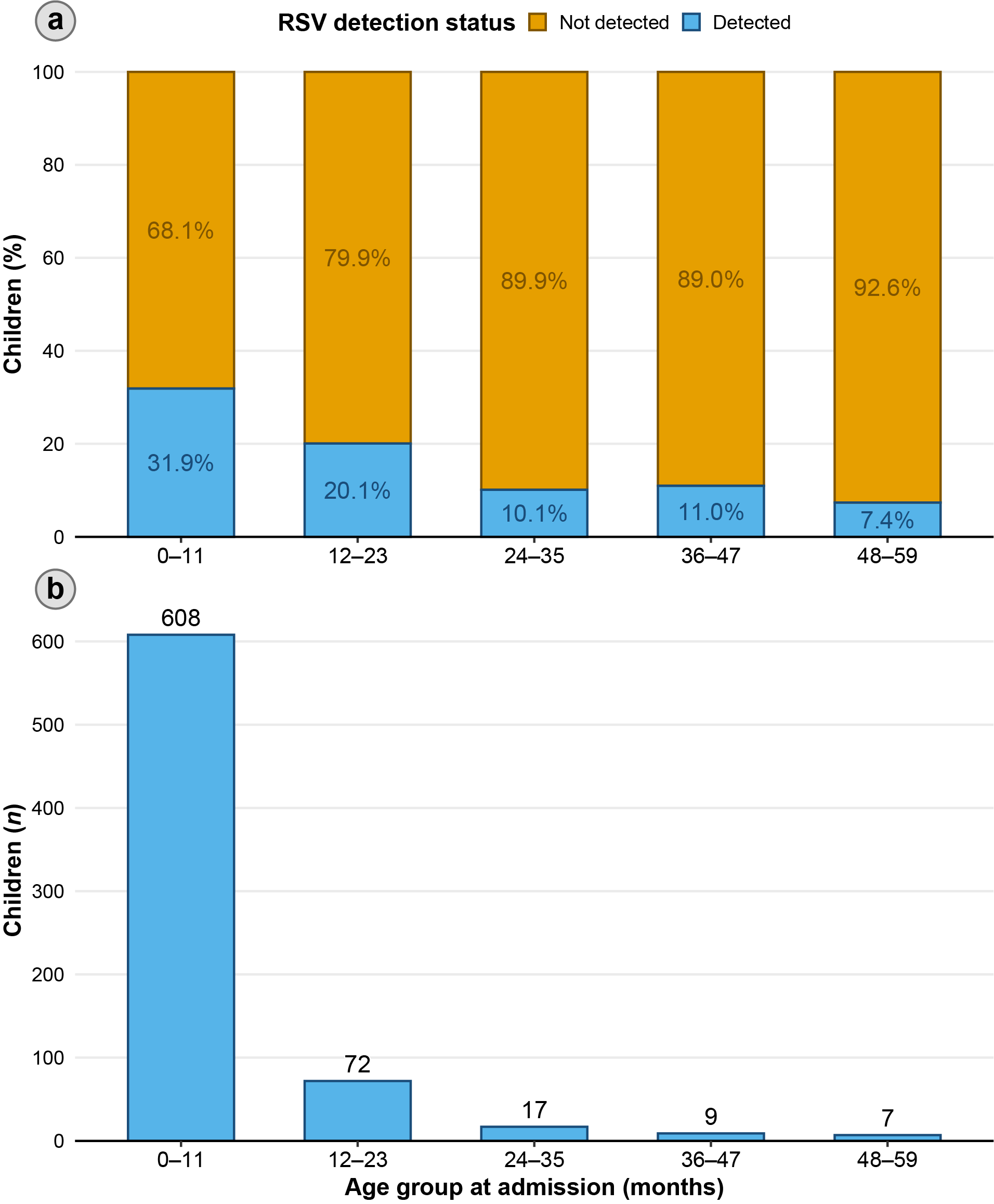


## Jordan Viral Surveillance Studies Group

**Al-Bashir Hospital:** Ahmad Alayyat; Yasmeen Owesi

**Eastern Mediterranean Public Health Network:** Lara Kufoof

**The University of Jordan:** Hanan H. Amin; Nader Alaridah

**Vanderbilt University Medical Center:** Marcia A. Blair; Rendie McHenry; Wanderson Rezende
